# Multidirectional Effects of Terpenoids from *Sorbus intermedia* (EHRH.) PERS Fruits in Cellular Model of Benign Prostate Hyperplasia

**DOI:** 10.3390/ph16070965

**Published:** 2023-07-05

**Authors:** Agnieszka Sołtys, Agnieszka Galanty, Karolina Grabowska, Paweł Paśko, Paweł Zagrodzki, Irma Podolak

**Affiliations:** 1 Department of Pharmacognosy, Faculty of Pharmacy, Jagiellonian University Medical College, Medyczna 9, 30-688 Kraków, Poland; agnieszka.soltys@doctoral.uj.edu.pl (A.S.); karolina1.grabowska@uj.edu.pl (K.G.); irma.podolak@uj.edu.pl (I.P.); 2 Department of Food Chemistry and Nutrition, Faculty of Pharmacy, Jagiellonian University Medical College, Medyczna 9, 30-688 Kraków, Poland; p.pasko@uj.edu.pl (P.P.); pawel.zagrodzki@uj.edu.pl (P.Z.)

**Keywords:** *Sorbus intermedia*, terpenoids, benign prostatic hyperplasia, antiproliferative activity, antiandrogenic activity, anti-inflammatory activity, fruits

## Abstract

Benign prostatic hyperplasia (BPH) is a common urological disease affecting aging men. Its pathogenesis is regarded as complex and multifactorial, with sex hormones and inflammation as key contributory factors. In the current study, we investigated the anti-BPH potential of terpenoids present in the fruits of *Sorbus intermedia* (EHRH.) PERS. Not only the effects on testosterone-stimulated normal prostate epithelial PNT2 cells, namely suppression of 5-*α*-reductase activity, PSA secretion, and cell proliferation, were determined but also the inhibitory activity on heat-induced protein denaturation, hyaluronidase, as well as IL-6, TNF-α, and NO release in LPS-treated macrophages. *Sorbus* terpenoids significantly inhibited 5-*α*-reductase activity and reduced PSA secretion in PNT2 cells, reversing the stimulatory effect of testosterone. PNT2 cell proliferation was also found to be attenuated. Subsequently, all compounds reduced the release of pro-inflammatory mediators in RAW 264.7 cells. In addition, ursolic acid (UA) and its aldehyde (UAL) were the most potent hyaluronidase inhibitors of all compounds, with IC_50_ values of 225.75 µg/mL and 369.77 µg/mL, respectively. For better understanding and interpretation of the overall effect of *Sorbus* terpenoids on different aspects of BPH pathogenesis and development, cluster analysis was applied.

## 1. Introduction

Benign prostatic hyperplasia (BPH) is a non-malignant enlargement of the prostate gland. It is a very common urological disease affecting aging men. Histopathologically, it is manifested by an increase in the number of cells of the epithelial and fibromuscular tissues of the transition zone of the prostate gland and the periurethral region [1].

The aetiology and pathophysiology of BPH are still not fully understood. Several theories of its origin and development have been proposed [2,3], and various permissive factors have been indicated to date [1,4]. The role of sex hormones and chronic inflammation are highly disputed. Animal and human studies suggest a significant role of dihydrotestosterone (DHT), which is the main androgen of the prostate [5]. As for chronic inflammation, it is also thought to play an important role in the development of BPH, particularly in the occurrence, progression, and severity of clinical symptoms associated with BPH [1,5,6,7].

Although BPH is not life-threatening, pathological changes within the prostate gland often affect urinary function, leading to clinical symptoms known as LUTS (lower urinary tract symptoms), which are mainly related to urination dysfunction. However, it should be noted that not all men experience LUTS, and a relatively low correlation between prostate volume, urinary tract symptoms, and urinary flow rate has been observed [8,9]. Recent studies have shown that the lifetime prevalence of BPH-associated LUTS is estimated at 26.2% worldwide and increases from 14.8% to 36.8% in men aged 40 and ≥80, respectively [10]. Nevertheless, urinary symptoms cause discomfort and inconvenience in performing daily activities and significantly reduce quality of life [5]. Furthermore, as BPH is closely associated with age, the incidence and prevalence rates of this disorder are expected to increase in an aging population, and this expected burden will challenge health care systems [8,11].

Current treatments of BPH include watchful waiting, pharmacotherapy (mainly 5-*α*-reductase inhibitors and *α*1-blockers), phytotherapy, and several forms of active intervention, such as various surgical and non-surgical procedures [8,9]. The appropriate therapy is selected on the basis of the clinical manifestation of the disease as well as the patients’ health status and individual preferences [8].

Herbal medicines are very popular among patients and administered in mild to moderate BPH. It is also not without significance that they are easily available and usually well tolerated. They also appear to be suitable for prevention, as BPH is characterized by high morbidity and a long latency period [3]. Several modes of action of natural products used in BPH have been proposed, including antiandrogenic, antiproliferative, and anti-inflammatory activity, but most studies refer to the activity of herbal extracts [2,3]. Some studies point to triterpenes as potential anti-BPH agents and, although the literature is scarce, provide encouraging results [12,13,14,15,16,17].

Recently, we reported on isolation of ursane terpenoids, i.e., ursolic acid (UA), ursolic aldehyde (UAL), 3-*O*-*β*-acetoxy-ursolic acid (AUA), 3-*O*-*β*-acetoxy-19*α*-hydroxy-ursolic acid (AHUA), and uvaol (UO), as well as *β*-sitosterol (*β*SIT), from fruits of *Sorbus intermedia* (EHRH.) PERS (Rosaceae), which is a popular ornamental tree native to northern Europe [18]. Some of these compounds were found to exhibit cytotoxic activity against, among others, human prostate cancer cell lines DU145 and PC3, with almost null toxic effects on normal prostate epithelial PNT2 cells. Satisfactory results against PC3 cells after 48 h of incubation were obtained for UA, AUA, and UAL, with IC_50_ values of 4.45 µg/mL, 16.40 µg/mL, and 22.45 µg/mL, respectively [18]. Previously, chloroform extracts also prepared from *S. intermedia* fruits at different developmental stages were found to reduce the viability of prostate cell lines [19]. This prompted us to extend our investigations to another prostate-related health problem, namely BPH. Therefore, the aim of the current study was to further investigate terpenoids isolated from *S. intermedia* with regard to their anti-BPH potential. Given the complexity of BPH pathogenesis, these compounds have been subjected to a number of in vitro studies to assess their multidirectional effects on different aspects of BPH. We designed and proposed an in vitro model based on testosterone-stimulated PNT2 cells and assessed whether these compounds could affect 5-*α*-reductase activity, PSA release, and cell hyperproliferation. We then determined the anti-inflammatory properties of all compounds. Finally, we applied hierarchical agglomeration cluster analysis to investigate the similarities between the activities of these compounds in the different experiments performed in this work to better interpret their overall effect in the human BPH model and inflammation and possibly identify the most promising multi-target compounds.

## 2. Results and Discussion

### 2.1. Antiandrogenic Effects of S. intermedia Terpenoids on Testosterone-Stimulated PNT2 Cells

Dihydrotestosterone (DHT) is the main androgen of the prostate. More than 90% of testosterone is converted in stromal cells to the more active DHT by 5-*α*-reductase. Moreover, the prostate remains sensitive to androgens throughout life, and simultaneously, intraprostatic DHT levels remain high despite aging [20]. Animal and human studies suggest its significant involvement in the development of BPH, as DHT is believed to play a key role in maintaining homeostasis between cell proliferation and apoptosis [5,20]. DHT binds, with higher affinity than testosterone, to the androgen receptor, which ultimately promotes transcription of androgen-dependent genes and further protein synthesis, differentiation, as well as cell growth. Moreover, androgens act indirectly by stimulation of production of several growth factors, e.g., EGF (epidermal growth factor), KGF (keratinocyte growth factor), and IGFs (insulin-like growth factors), involved in cell proliferation [3,5,20]. The prostatic stroma and epithelium interact through cell signalling mechanisms mediated by DHT and DHT-stimulated growth factors. DHT influences prostate cells in both an autocrine and paracrine manner [5]. For this reason, 5-*α*-reductase has become a pharmacological target in the treatment of BPH, and subsequently, inhibitors of this enzyme, such as dutasteride and finasteride, were introduced to the therapeutics. These drugs effectively reduce DHT levels and prostate volume; however, they are burdened with some bothersome side effects, such as decreased libido and impotence.

**Figure 1:**
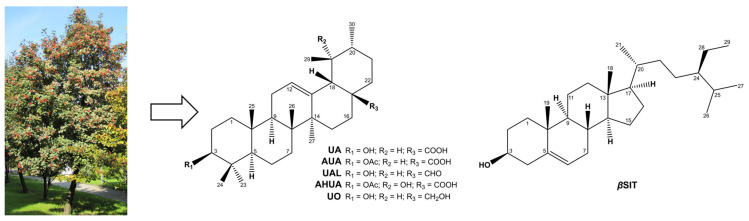
Chemical structures of terpenoids isolated from *S. intermedia* fruits [18].

**Figure 2:**
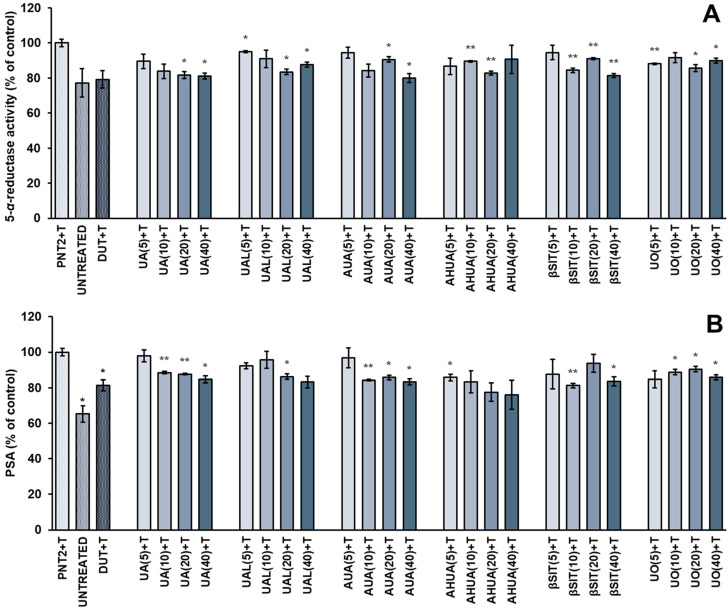
The effects of *S intermedia* terpenoids on 5-*α*-reductase activity (**A**) and PSA secretion (**B**) in PNT2 cells stimulated by testosterone. PNT2 cells were incubated with terpenoids at different concentrations (the numbers in parentheses indicate concentrations in µg/mL) in the presence of testosterone (T). The results are expressed as the mean ± SD of three experiments. The results are set together with untreated PNT2 cells (UNTREATED) and cells treated with testosterone and dutasteride as the reference drug (DUT+T). Statistical analysis was performed using one-way ANOVA and T3 Dunnett post hoc test with * *p* < 0.05, ** *p* < 0.01, against the testosterone-stimulated cells. Abbreviations: UA, ursolic acid; UAL, ursolic aldehyde; AUA, 3-*O*-*β*-acetoxy-ursolic acid; AHUA, 3-*O*-*β*-acetoxy-19*α*-hydroxy-ursolic acid; *β*SIT, *β*-sitosterol; UO, uvaol.

On this basis, we investigated the effect of natural terpenoids (Figure 1) from *S. intermedia* on 5-*α*-reductase activity in testosterone-stimulated PNT2 cells. Although the post hoc analysis showed no significance between the control groups in this study, there is a strong and obvious trend that clearly indicates a stimulating effect of the hormone on prostate cells. We also measured PSA (prostate-specific antigen) secretion, as the PSA gene (*KLK3*) is regulated by androgens, and PSA serves as biomarker for prostate diseases such as prostate cancer and BPH [21]. The results of our experiments are shown in Figure 2. In this study, we used dutasteride as a reference drug. It is one of the 5-*α*-reductase inhibitors that is indicated in moderate to severe BPH.

All *Sorbus* compounds tested, including ursane derivatives and *β*-sitosterol, significantly inhibited 5-*α*-reductase activity and reduced PSA secretion in testosterone-stimulated PNT2 cells. UA, AUA, and *β*SIT showed suppression of the enzyme activity by approximately 20% at a concentration of 40 µg/mL, which was comparable to the reference drug. Both dutasteride and *Sorbus* compounds reversed the stimulatory effect of testosterone on 5-*α*-reductase activity. In addition, all compounds reduced PSA release in PNT2 cells to 76–86% at the highest concentrations, and there were no statistically significant differences between tested compounds and dutasteride. Although we did not observe a strict dose–effect relationship, some compounds showed a tendency to act in dose-dependent manner, especially UA, UAL, and AUA.

Of the *Sorbus* compounds tested in the current work, only *β*SIT has been studied more extensively, including by in vivo human studies, as a potential anti-BPH agent. Systematic reviews indicate that *β*SIT improves urinary symptoms but does not reduce prostate size [22,23]. Previously, *β*SIT was found to reduce 5-*α*-reductase activity in the hamster prostate in a dose-dependent manner [24]. In addition, there is evidence for the antiandrogenic activity of several herbs used in the treatment of BPH, such as *Serenoa repens*, *Pygeum africanum*, and *Urtica dioica*, whose main active constituents are sterols [25,26]. Interestingly, a standardized saw palmetto extract enriched in *β*SIT was recently studied, and no significant inhibitory activity on 5-*α*-reductase was observed [27].

To the best of our knowledge, literature reports on the anti-BPH potential of ursane-based triterpenes are extremely scarce. One study in a rat model of testosterone-induced BPH showed that treatment with ursolic acid resulted in a reduction of prostate volume as well as in serum and prostate tissue DHT levels [12]. Interestingly, an isomer of ursolic acid–oleanolic acid downregulated 5-*α*-reductase II expression in a rat model of BPH while inhibiting prostate growth and serum DHT levels, and the results were comparable to finasteride [17].

### 2.2. Antiproliferative Activities of S. intermedia Terpenoids 

**Figure 3:**
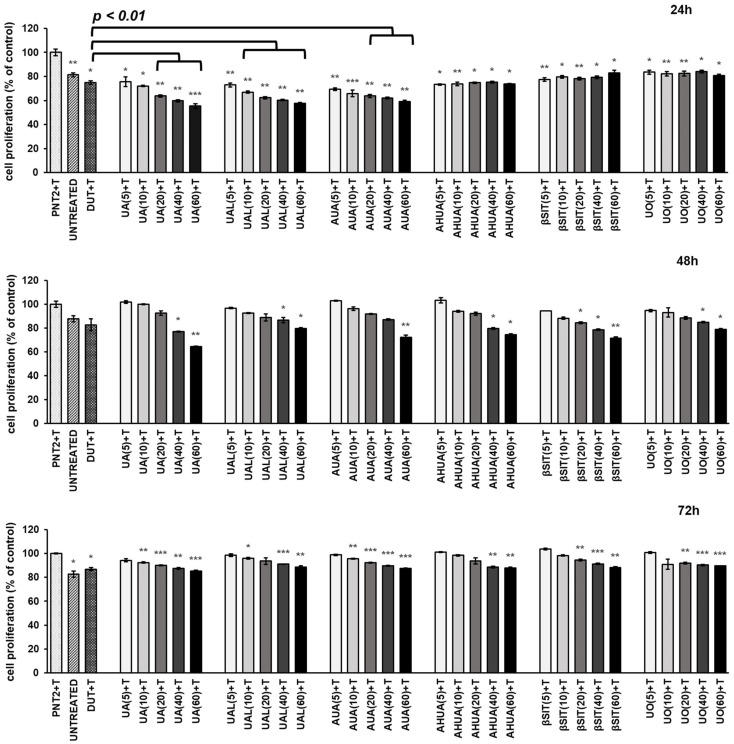
The anti-proliferative effects of *S intermedia* terpenoids on testosterone-stimulated PNT2 cells. PNT2 cells were incubated with terpenoids at different concentrations (the numbers in parentheses indicate concentrations in µg/mL) in the presence of testosterone (T). The results obtained after 24 h, 48 h, and 72 h of incubation are presented separately on each bar chart and expressed as the mean ± SD of three experiments. The results are set together with untreated PNT2 cells (UNTREATED) and cells treated with testosterone and dutasteride as the reference drug (DUT+T). Statistical analysis was performed using one-way ANOVA and T3 Dunnett post hoc test with * *p* < 0.05, ** *p* < 0.01, and *** *p* < 0.001 against the testosterone-stimulated cells. Significant differences (*p* < 0.01) between tested compounds and reference drug are marked by black line. Abbreviations: UA, ursolic acid; UAL, ursolic aldehyde; AUA, 3-*O*-*β*-acetoxy-ursolic acid; AHUA, 3-*O*-*β*-acetoxy-19*α*-hydroxy-ursolic acid; *β*SIT, *β*-sitosterol; UO, uvaol.

The development of BPH is related to excessive and abnormal cell proliferation. Pathological hypertrophy of prostate gland is considered to be associated with the imbalance between cell proliferation and apoptosis, which is modulated predominantly by prostatic androgens. Therefore, we investigated the antiproliferative activity of *S. intermedia* terpenoids and *β*SIT towards testosterone-treated PNT2 cells. Effects were observed after 24 h, 48 h, and 72 h and were compared with dutasteride, which was used as a reference drug. The results are shown in Figure 3.

All compounds reduced cell proliferation, with better results obtained after 24 h and 48 h of incubation. With the exception of the results obtained after 24 h for AHUA, *β*SIT, and UO, all other compounds supressed or tended to supress cell proliferation in a dose-dependent manner. UA, UAL, and AUA were more potent than the reference drug, causing an approximately 45% decrease in cell proliferation at a concentration of 60 µg/mL after 24 h. Although their cytostatic effect was less pronounced after 72 h of incubation, they were still as active as the reference drug.

UA was previously reported to exert cytotoxic [18,28,29,30] or cytostatic [31] activities against various prostate cell lines. For example, a 24 h incubation of PC3 cells with 40 μM of UA resulted in a significant increase in the number of cells in the G1 phase [31]. Its combination at a low dose (4.1 μM) with oleanolic acid (5.47 μM) induced cytotoxic autophagy and inhibited the growth of BPH-1 cells by 50%, which proved more effective than administration of UA alone (50% growth inhibition at a concentration of 67 μM) [16]. In addition, UA arrested the cell cycle in several other cells of non-prostatic origin [32,33,34,35]. Similarly, the antiproliferative activity of *β*SIT against cells of various cancer types is quite well documented [36], including its activity towards PC3 cells, where a 1-day incubation with 16 μM of *β*SIT led to a 35% growth inhibition [37].

For other *Sorbus* terpenoids, i.e., UAL, AUA, AHUA, and UO, there is a lack of information in the literature, especially with regard to their effects on prostate cells. In a previous work, we already determined their cytotoxic effects against DU145, PC3, and PNT2 cells using the lactate dehydrogenase assay, which indicates a cell death. AHUA and UO showed no effects on all cells tested, while UAL and AUA selectively affected the viability of DU145 and PC3 cells without toxicity towards normal PNT2 cells [18]. Nevertheless, to the best of our knowledge, there is no other report relating to the antiproliferative activity of these compounds on any cells of prostatic origin. With regard to non-prostatic cell lines, AUA and UO inhibited the growth of A375 [38] as well as HepG2 and MCF-7 cells [39,40], respectively, while UAL, in a mixture with oleanolic aldehyde, exerted an antiproliferative effect on MCF-7 cells with GI_50_ value equal to 202 µM [41]. To the best of our knowledge, no similar studies on in vitro hyperproliferation of prostate cells were found for any of the terpenoids tested.

### 2.3. Anti-Inflammatory Activities of S. intermedia Terpenoids 

The impact of inflammation on the development of BPH appears to be complex and reciprocal, as inflammation can not only promote the development of BPH alone or in interaction with sex hormones, but it can also be induced during BPH, thus leading to expansion of its severity [7]. Several studies have found that chronic inflammation is associated with higher prostate volume, IPSS (international prostate symptom score), acute urinary retention, and/or higher risk of prostatectomy [42]. Therefore, we decided to assess the anti-inflammatory potential of *S. intermedia* compounds.

#### 2.3.1. Inhibition of Albumin Heat-Induced Denaturation

As part of anti-inflammatory experiments, *Sorbus* ursane terpenoids and *β*SIT were first screened for their ability to stabilize serum albumin against heat-induced denaturation, as protein denaturation is considered part of the inflammation process. Non-steroidal anti-inflammatory drugs, e.g., indomethacin or diclofenac sodium, were found to exhibit anti-denaturation effect and stabilize serum albumin. In our study, however, most of the compounds tested failed to inhibit heat-induced denaturation of albumin, and only AHUA and UO (Table 1) slightly protected the protein, showing approximately 30% inhibition in the concentration range of 25–1000 µg/mL and 100–1000 µg/mL, respectively. The results were compared to diclofenac sodium, which was used as a reference drug.

#### 2.3.2. The Effects of *S. intermedia* Terpenoids on the Release of Pro-Inflammatory Mediators in LPS-Stimulated RAW 264.7 Macrophages

Robert et al. (2009) showed that more than 80% of surgically treated patients had CD8 T-lymphocyte and macrophage infiltrates in prostate tissues [43]. Some have identified BPH as an autoimmune disease and TNF-*α* as a potential therapeutic target, pointing to the ability of TNF antagonists to reduce epithelial hyperplasia, NFκB activation, and macrophage-mediated inflammation [44,45]. A retrospective study indicated that prostate tissues from TNF-antagonist-treated patients had a lower level of inflammation when compared to untreated group [44]. Tong et al. (2022) attempted to investigate the relationship between DHT and inflammation in BPH development and progression. The authors revealed that DHT stimulates the prostate stromal cells’ proliferation by increasing TNF-*α* expression in LPS-stressed M1 macrophages. Moreover, they analysed tissues from BPH patients and found that TNF-α expression was increased in patients with larger prostate volume [46]. Subsequently, elevated levels of other pro-inflammatory cytokines, including IL-6, IL-8, and IL-17, were found in BPH tissues [16]. A higher risk of BPH is also linked to metabolic syndrome, in which increased levels of C-reactive protein, IL-1*β*, IL-6, IL-8, and TNF-*α* were observed [42].

**Figure 4:**
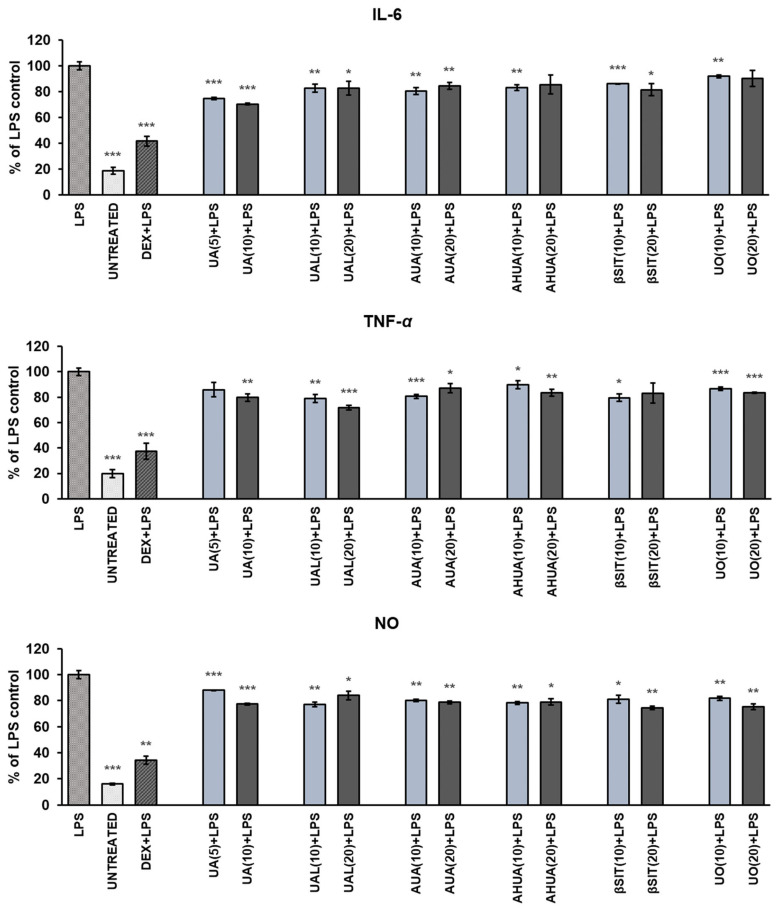
The impact of *S intermedia* terpenoids on the release of IL-6, TNF-α, and NO in LPS-stimulated RAW 264.7 macrophages. RAW cells were pre-treated with terpenoids at different concentrations (the numbers in parentheses indicate concentrations in µg/mL) for 1 h, followed by the addition of 10 ng/mL of LPS to induce inflammation. Exceptionally, due to high cytotoxicity towards RAW cells, UA were analysed at lower concentrations. Values are presented as the mean ± SD of three experiments. The results are set together with untreated RAW cells (UNTREATED) and cells treated with LPS and dexamethasone as the reference drug (DEX+LPS). Statistical analysis was performed using one-way ANOVA and T3 Dunnett post hoc test with * *p* < 0.05, ** *p* < 0.01, and *** *p* < 0.001 against the LPS-stimulated cells. Abbreviations: UA, ursolic acid; UAL, ursolic aldehyde; AUA, 3-*O*-*β*-acetoxy-ursolic acid; AHUA, 3-*O*-*β*-acetoxy-19*α*-hydroxy-ursolic acid; *β*SIT, *β*-sitosterol; UO, uvaol.

Hence, we decided to test whether compounds isolated from fruits of *S. intermedia* are able to influence the release of selected pro-inflammatory mediators in LPS-stimulated RAW 264.7 macrophages. The results are shown in Figure 4. In this assay, dexamethasone, a glucocorticoid with an anti-inflammatory properties, was used as a reference drug.

All compounds at sub-cytotoxic concentrations significantly reduced the release of all tested mediators in LPS-stimulated RAW 264.7 macrophages. The strongest suppression of IL-6 release was observed with UA (about 30% at concentration of 10 µg/mL), and this effect was dose-dependent (*p* < 0.001). UAL was most effective towards TNF-*α* and reduced its secretion up to 72% at a concentration of 20 µg/mL. *Sorbus* terpenoids reduced NO release in LPS-stimulated RAW cells comparably by approximately 20–25% at their most effective concentrations. A strong dose–effect relationship (*p* < 0.001) was observed only for UA.

Biological studies on the anti-inflammatory activity of UAL and AHUA are extremely unaddressed. Only UA and UO are reasonably well examined. Du et al. (2020) showed that in LPS-treated macrophages, UO significantly suppressed NO production as well as mRNA expression and secretion of several mediators, including IL-6 and TNF-*α* [47]. Significant reductions in NO production at concentrations of 3 μM, 10 μM, and 30 μM were also reported by Wang et al. (2020), but 50% inhibitory activity was not achieved [48]. UO was also considered to lack activity against NO production in LPS and IFN-γ -treated [49] and LPS-stressed RAW 264.7 cells, with IC_50_ above 100 μM [50]. With regard to UA, several studies indicate its inhibitory activity against NO production in stimulated macrophages [49,51,52]. In contrast, in the study of Zhou and Wink (2019), UA did not cause a statistically significant decrease in NO level in RAW264.7 cells at the concentration range of 3–9 µM; however, it downregulated the TNF-*α* expression [53]. In one study, UA and AUA suppressed the expression and production of TNF-*α* and IL-6 in TNF-α-stimulated RA synovial fibroblasts [54]. *β*SIT was also previously reported to reduce TNF-*α* and IL-6 production in LPS-stimulated RAW 264.7 cells [55]. However, it was also regarded as inactive against NO secretion in LPS and IFN-γ-treated RAW cells with an IC_50_ above 100 μM [48].

#### 2.3.3. The Effects of *S. intermedia* Terpenoids on Hyaluronidase Activity

The extracellular matrix is a network composed of macromolecules such as collagens, elastin, proteoglycans/glycosaminoglycans (e.g., hyaluronic acid), and different glycoproteins [56]. It not only serves as a structural scaffold but also contributes to several cellular processes, such as proliferation, migration, differentiation, autophagy, and angiogenesis. For example, hyaluronic acid and its degradation products interact with CD44 in a size-dependent manner. Long-chain hyaluronic acid induces receptor clustering, while low-mass molecules induce various signalling pathways involved in the regulation of cytoskeletal organization, cell growth, and proliferation [57].

Because matrix components have such important regulatory functions, the enzymes involved in their catabolism are essential in maintaining homeostasis. Indeed, increased hyaluronic acid degradation, catalysed by hyaluronidases, is observed in several pathophysiological processes [58]. The role of hyaluronidase in BPH is unknown, but there are reports linking the activity of this enzyme to the development and progression of prostate cancer [59,60]. It is also worth noting that hyaluronidases leading to increased tissue permeability are recognized as factors that spread inflammation.

Therefore, we examined the hyaluronidase inhibitory potential of compounds isolated from *S. intermedia* using the turbidimetric method and quercetin as a reference substance. The results are shown in Table 2. *β*SIT and UO were found to be practically inactive with IC_50_ > 1000 µg/mL, but UA and UAL were more potent than the reference (quercetin, IC_50_ = 517.05 µg/mL), with IC_50_ values 225.75 µg/mL and 369.77 µg/mL, respectively. AHUA exerted similar activity to quercetin. Interestingly, AUA and *β*SIT slightly inhibited the enzyme at lower concentrations.

To the best of our knowledge, this is the first report on the anti-hyaluronidase activity of UAL, AHUA, and UO. It was previously reported that *β*SIT inhibits hyaluronidase, with IC_50_ = 888.5 ± 44.9 µg/mL [61]. In another study, it suppressed the enzyme activity by approximately 32% at a concentration of 100 µg/mL [62]. These results are in agreement with ours. Meanwhile, the literature data on ursolic acid activity are divergent, ranging from 40–60% inhibition at a concentration of 1000 µg/mL [63,64] to about 50% suppression of the enzyme at a concentration range from 25 to 50 µg/mL [65]. Our results best match those obtained by Michel et al. (2017), who determined the IC_50_ value for this compound equals to 380.14 ± 10.92 µg/mL [66]. In contrast to our study, Abdullah et al. (2016) previously reported higher UA and AUA activities with IC_50_ values equal to 103.18 ± 1.70 µM and 136.92 ± 0.04 µM, respectively [67]. Such discrepancies in results clearly demand more in-depth studies.

### 2.4. Chemometric Analysis

Hierarchical agglomeration cluster analysis (CA) was used to investigate the similarity between the compounds tested and thus better understand and interpret their overall and multidirectional effects on the human BPH cellular model and inflammation. Results obtained for six compounds tested at two different concentrations, 10 and 20 µg/mL, respectively, were included, as only these concentrations gave results for all parameters. These parameters were as follows: antiproliferative effects after 24 h, 48 h, and 72 h of incubation; 5-*α*-reductase inhibitory activity; PSA secretion; effect on release of IL-6; TNF-α and NO in LPS-stimulated macrophages; anti-hyaluronidase activity; and inhibition of albumin denaturation. Exceptionally, for studies on LPS-stimulated macrophages, results obtained for UA at concentrations of 5 µg/mL and 10 µg/mL were analysed.

**Figure 5:**
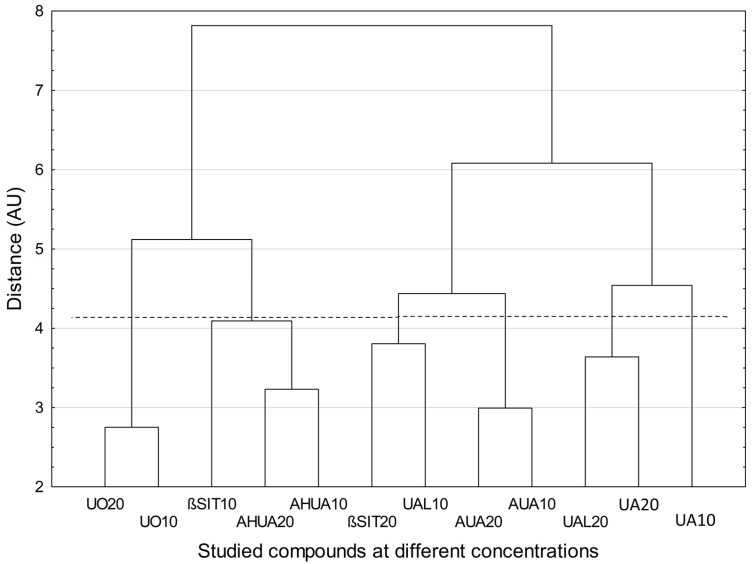
Dendrogram of similarity among investigated chemical compounds at different concentrations (function of the distance: Euclidean distance; method of grouping: Ward method; the added dashed horizontal line indicates that the grouping has been stopped according to the Mojena’s rate). Numbers indicate concentrations in µg/mL. Exceptionally, in case of studies on LPS-stimulated macrophages, the results obtained for UA at concentrations of 5 µg/mL and 10 µg/mL were included and assigned as UA10 and UA20, respectively. Abbreviations: UA, ursolic acid; UAL, ursolic aldehyde; AUA, 3-*O*-*β*-acetoxy-ursolic acid; AHUA, 3-*O*-*β*-acetoxy-19*α*-hydroxy-ursolic acid; *β*SIT, *β*-sitosterol; UO, uvaol.

Cluster analysis showed specific similarity for two compounds (UO and AUA) at two concentrations (10 and 20 µg/mL) (Figure 5). The samples corresponding to these compounds at these concentrations formed two separate two-element clusters, with no other samples. A third such cluster occurred for compound AHUA but was below the Mojena’s criterion. In the remaining cases, mixed clusters (containing samples of different compounds at different concentrations) were found. Overall, UO, *β*SIT and AHUA are distinctly distant from UA, UAL, and AUA regardless of the concentration at which they were used, indicating the differences between the effects that these compounds displayed.

## 3. Materials and Methods

### 3.1. Reagents and Instruments

Dulbecco’s Modified Eagle’s Medium F12 HAM (DMEM/F12), Dulbecco’s Modified Eagle’s Medium with 4500 mg/L glucose (DMEM high glucose), foetal bovine serum (FBS), phosphate-buffered saline (PBS), crystal violet, formaldehyde, lipopolysaccharide (LPS), DMSO, albumin from bovine serum (BSA): fraction V ≥ 98% (A3294), hyaluronidase from bovine testes type I-S, *Streptococcus equi* hyaluronic acid (HA), cetyltrimethylammonium bromide (CTAB), quercetin dihydrate, ursolic acid (other terpenoids were obtained by isolation), diclofenac sodium, dutasteride, and testosterone propionate were obtained from Sigma-Aldrich (Seelze, Germany). Acetate buffer pH 4.5 was purchased from J.T. Baker Chemical Co. (Phillipsburg, NJ, USA).

### 3.2. Cell Culture Conditions

Experiments were performed on human PNT2 prostate epithelial cells (ECACC 95012613, Merck, Darmstadt, Germany). For anti-inflammatory assay, murine RAW264.7 macrophages were used. The cells were cultured in a humidified atmosphere with 5% CO_2_ at 37 °C in DMEM/F12 (PNT2) or DMEM high glucose (RAW 264.7) supplemented with 10% (FBS), 100 IU/mL penicillin, and 10 µg/mL streptomycin. The tested compounds were diluted in the culture media from freshly made stock solution (10 mg/mL in acetone) to the working concentrations.

### 3.3. Determination of PSA and 5-α-reductase 

The experiment was performed according to Nakayama et al. (2021) [68]. Briefly, PNT2 cells were seeded onto 96 multi-well plates (1.5 × 10^5^ cells/well) for 24 h and then treated with the tested compounds at the concentrations of 5, 10, 20, and 40 µg/mL for 72 h. Dutasteride (10 µM) was used as a reference drug. Cell culture supernatants were collected and used for quantitative analysis of PSA and 5-*α*-reductase level, which was performed using a Human ELISA kits, according to the manufacturer’s protocol. The analyses were performed in triplicates, and the absorbance was measured using a microplate reader (SynergyTM HT—BioTek, Winooski, VT, USA). The results were determined as % of control.

### 3.4. Proliferation Assay

The cells were seeded onto 96-well plates (1 × 10^3^ cells/well) and incubated for 24 h. Then, the medium was replaced with fresh medium containing 0.5 μM of testosterone propionate (T) in order to stimulate cell proliferation, as observed in prostate hyperplasia, and the tested compounds were added. Dutasteride (10 µM) was used as a reference drug. After 24, 48, and 72 h of incubation, the cell number was determined using crystal violet assay, as described previously [69]. Briefly, the cells were washed with PBS and fixed with 3.7% formaldehyde. Then, crystal violet solution was added for 10 min, followed by washing with PBS. Crystal violet was extracted from cells using 1.33% citric acid and 1.09% sodium citrate in water/methanol (1:1) solution. The absorbance was measured at 570 nm. The proliferation rate was determined as a % of control.

### 3.5. Inhibition of Albumin Denaturation

The protective effects of compounds against albumin heat-induced denaturation were determined as was described previously [70]. Tested compounds were dissolved in DMSO and tested at concentrations from 10 to 1000 μg/mL. Briefly, examined substances were preincubated in 25 °C for 15 min in the presence of BSA. Next, the reaction mixtures were incubated in 70 °C for 5 min for proteins denaturation. After cooling the samples, the turbidity was measured at 660 nm using microplate reader (SynergyTM HT—BioTek). Diclofenac sodium was used as reference drug. The product control solution was prepared to diminish the sample background, and the absorbance of the medium was performed as a blind control of experiment. All assays were conducted in triplicate. The percent of inhibition of protein denaturation was calculated as follows:

% inhibition = 100 − [(AS − APc)/(AC − AB)] × 100

AS—absorbance of the tested substance;
APc—absorbance of the product control solution;
AC—absorbance in the absence of inhibitor;
AB—absorbance of blind control.

The half-maximal inhibitory concentration value IC_50_ was determined.

### 3.6. Determination of NO, IL-6 and TNF-α Release 

Prior to the anti-inflammatory experiments, the toxicity of the tested compounds to RAW 264.7 macrophages was determined. The cells were seeded onto 96 multi-well plates (1.5 × 10^5^ cells/well) and incubated with the tested compounds (0–100 µg/mL) for 24 h. Next, cell viability was tested with the MTT assay. All analyses were performed in triplicate, and the results are expressed as % of cell viability (mean ± SD). For further anti-inflammatory assay, the concentrations of 5 and 10 µg/mL for UA and 10 and 20 µg/mL for the other compounds were chosen as nontoxic.

For anti-inflammatory assays, RAW 264.7 cells were seeded onto 96 multi-well plates (1.5 × 10^5^ cells/well) and pre-treated with the tested compounds for 1 h, followed by the addition of 10 ng/mL of LPS to induce inflammation process, as described previously [69]. Dexamethasone (0.5 µg/mL) was used as a reference drug. The incubation was continued for the next 24 h. Cell culture supernatants were used for further analysis. The nitric oxide level was determined using Griess Reagent Kit (Promega Corporation (Madison, Winooski, VT, USA), according to the manufacturer’s protocol. The cytokine (TNF-α, IL-6) release level was performed using Human ELISA kits (Bioassay Technology Laboratory, Shanghai, China), according to the manufacturer’s protocol. The analyses were performed in triplicates, and the absorbance was measured using a microplate reader (SynergyTM HT—BioTek). The results were determined as % of control.

### 3.7. Anti-Hyaluronidase Assay

Hyaluronidase inhibitory activity was evaluated on 96-well microplates using turbidimetric method as we described previously [70]. Compounds were dissolved in DMSO and tested at concentration range from 10 to1000 µg/mL. Briefly, substances were preincubated at 37 °C for 10 min with the presence of incubation buffer, enzyme, and acetate buffer (pH 4.5). Then, HA was added, and incubation continued for further 45 min. In the next step, a CTAB solution was added to precipitate undigested HA. The amount of undigested HA is proportional to the turbidity; thus, enzymatic activity was quantified spectrophotometrically at 600 nm using microplate reader (SynergyTM HT—BioTek). The absorbance in the presence of enzyme and substrate (control I) and in the absence of enzyme (control II) was measured. Product control solution, with HA instead of buffer, was prepared to deduct the sample background. The absorbance of the medium was performed as a blind control of the experiment. All experiments were conducted in triplicate. Quercetin was used as reference substance. The inhibition in percentage was calculated using the following formula:

% inhibition = {[As − (APc − AB)] − AI }/{[AII − (APc – AB)] − AI } × 100

AI—absorbance of enzyme + substrate (control I);
AII—absorbance in the absence of enzyme (control II);
As—absorbance of sample solution;
APc—absorbance of the product control solution;
AB—absorbance of a blank control of experiment.

The half-maximal inhibitory concentration value IC_50_ was estimated.

### 3.8. Statistical Analysis

Comparison of means was carried out using IBM SPSS Statistics 29.0 for Windows. The data were analysed by one-way ANOVA followed by a T3 Dunnett post hoc test. The CA analysis was performed using Euclidean distance as a measure of distance between objects and Ward’s method of grouping objects. The number of clusters was set according to the Mojena’s rate. Prior to CA analysis, the data were standardized (z-transformed) to obtain zero mean and unit variance for each parameter. CA analysis was conducted by means of STATISTICA v.13.3. package (TIBCO Software Inc., Palo Alto, CA, USA). The same software was also used for the graphic representation of results.

## 4. Conclusions

In the current study*,* ursane-type triterpenoids and *β*-stitosterol isolated from the fruits of *Sorbus* were subjected to a series of in vitro experiments that were designed to assess their effects on various aspects of BPH pathogenesis and development. Not only did *Sorbus* compounds show antiandrogenic and antiproliferative effects on testosterone-treated PNT2 cells, reversing the stimulating effect of the hormone, but they also demonstrated an impact on inflammation, which is believed to play an important role in the development and progression of BPH and the clinical symptoms associated with BPH.

Considering the overall anti-BPH potential of *Sorbus* compounds, as shown by chemometric analysis, UA, UAL, and AUA formed a clearly distinct group from UO, *β*SIT, and AHUA, suggesting differences in the action of these compounds. Indeed, UA, UAL, and/or AUA tended to be among the most active compounds in the assays that were proposed and conducted in this study. Moreover, with the exception of *β*SIT, the examined *Sorbus* terpenoids were based on the ursane skeleton, i.e., UA and its derivatives. Thus, our study showed that chemical structure significantly influences activity. However, more targeted structure–activity studies are needed to better understand this relationship.

In conclusion, our study showed that *Sorbus* terpenoids exhibit antiandrogenic, antiproliferative, and anti-inflammatory properties and may represent an interesting target in the development of new anti-BPH therapies. Furthermore, the fruits of *S. intermedia* are edible and may be a valuable component of the daily diet.

## Figures and Tables

**Table 1:** The effects of *S. intermedia* terpenoids against heat-induced albumin denaturation.

Concentration	Albumin Denaturation Inhibition (%)		
(µg/mL)	AHUA	UO	DS
5	NE	NE	34.29 ± 2.07
10	19.61 ± 6.76	9.35 ± 2.50	52.45 ± 2.84
25	28.85 ± 2.50 *	11.17 ± 1.69 *	81.06 ± 3.04
50	31.46 ± 3.07 *	18.35 ± 5.37	95.75 ± 1.71
100	30.63 ± 3.34 *	28.49 ± 3.82 *	99.06 ± 0.26
250	22.79 ± 5.00	31.98 ± 2.34 *	99.76 ± 0.26
500	28.67 ± 2.72 *	30.78 ± 1.56 *	100.00 ± 0.00
750	30.24 ± 2.72 *	29.30 ± 1.28 *	100.00 ± 0.00
1000	32.81 ± 4.75 *	30.37 ± 3.36 *	100.00 ± 0.00
IC_50_	>Cmax	>Cmax	8.61

Inhibition of albumin denaturation was measured using turbidimetric assay. Values are presented as the mean ± SD of three experiments. Statistical analysis was performed using one-way ANOVA and T3 Dunnett post hoc test against positive control. * *p* < 0.05 indicates significant denaturation inhibition. UA, UAL, AUA, and *β*SIT were not active. Abbreviations: AHUA, 3-*O*-*β*-acetoxy-19*α*-hydroxy-ursolic acid; UO, uvaol; DS, diclofenac sodium; NE, not examined.

**Table 2:** Anti-hyaluronidase activity of *S. intermedia* terpenoids.

Concentration (µg/mL)	Hyaluronidase Inhibition (%)						
	UA	UAL	AUA	AHUA	*β*SIT	UO	QUERCETIN
10	0.00 ± 0.00	NE	7.87 ± 0.95 *	0.00 ± 0.00	3.72 ± 0.78 *	0.00 ± 0.00	0.61 ± 0.53
25	2.52 ± 2.18	0.00 ± 0.00	11.70 ± 1.55 *	0.17 ± 0.30	12.09 ± 0.28 *	0.00 ± 0.00	1.17 ± 0.41
50	3.23 ± 1.71	1.62 ± 0.35	16.76 ± 4.36	0.35 ± 0.30	20.53 ± 0.75 *	0.00 ± 0.00	1.18 ± 0.95
100	11.65 ± 0.92 *	3.91 ± 0.56	19.27 ± 4.85	2.32 ± 1.88	23.32 ± 0.75 *	0.00 ± 0.00	4.36 ± 0.88
250	60.31 ± 0.88 *	15.45 ± 1.67	30.57 ± 0.68 *	18.54 ± 3.44	25.79 ± 0.45 *	0.52 ± 0.52	18.08 ± 1.00
500	82.38 ± 3.77 *	78.79 ± 1.63 *	45.63 ± 1.37 *	42.07 ± 1.72	27.78 ± 3.83	4.01 ± 1.14	38.84 ± 1.33
750	85.35 ± 2.30	NE	52.13 ± 4.94	93.41 ± 2.75	30.56 ± 3.89	13.71 ± 1.53	87.27 ± 1.16
1000	96.01 ± 3.54	96.25 ± 0.86	56.73 ± 4.26	99.22 ± 0.68 *	36.75 ± 4.61	35.13 ± 4.85	90.94 ± 1.72
IC_50_	225.75	369.77	705.74	519.87	>Cmax	>Cmax	517.05

Hyaluronidase inhibition was measured using turbidimetric assay. Values are presented as the mean ± SD of three experiments. Statistical analysis was performed using one-way ANOVA and T3 Dunnett post hoc test with * *p* < 0.05 vs quercetin. Abbreviations: UA, ursolic acid; UAL, ursolic aldehyde; AUA, 3-*O*-*β*-acetoxy-ursolic acid; AHUA, 3-*O*-*β*-acetoxy-19*α*-hydroxy-ursolic acid; *β*SIT, *β*-sitosterol; UO, uvaol; NE, not examined.

## Data Availability

The data are contained within the article.
